# PPARα and RXRα in the regulation of neuronal ceroid lipofuscinosis genes: implications for Batten disease therapy

**DOI:** 10.1515/nipt-2026-0005

**Published:** 2026-04-28

**Authors:** Sujyoti Chandra, Kalipada Pahan

**Affiliations:** Department of Neurological Sciences, Rush University Medical Center, Chicago, IL, USA; Department of Neurological Sciences, Rush University Medical Center, 1735 West Harrison St, Suite 310, Chicago, IL 60612, USA; and Division of Research and Development, Jesse Brown Veterans Affairs Medical Center, 820 South Damen Avenue, Chicago, IL, USA

**Keywords:** Batten disease, CLN genes, PPARalpha, RXRalpha, therapeutic approach

## Abstract

Neuronal ceroid lipofuscinosis or Batten disease comprises a category of autosomal recessive neurodegenerative disorders that primarily affect children. Mutations in different genes lead to different forms of neuronal ceroid lipofuscinoses (CLN1–14). At present, there is no established therapy to cure most of the neuronal ceroid lipofuscinoses and the treatments are symptomatic. Enzyme replacement therapy, gene therapy, stem cell transplantation, and pharmacological chaperone therapy are being tested in different animal models and human patients. Peroxisome proliferator-activated receptor alpha (PPARα) is a member of the nuclear hormone receptor superfamily, which along with its transcription partner retinoid X receptor alpha (RXRα) regulates the expression of their target genes. This review highlights the potential role of PPARα and RXRα in the regulation of *CLN* genes. Here, using the MatInspector program of the Genomatix software, we performed promoter analyses of all *CLN* genes and observed that most of the *CLN* genes harbor one or more potential binding sites for PPAR and RXR in their promoter region. We further grouped them according to a binding prediction of the transcription factors to indicate high affinity binding of PPAR to *CLN2*, *CLN3*, *CLN4*, *CLN5, CLN7*, *CLN10*, *CLN11*, *CLN12*, and *CLN14*. On the other hand, we observed high affinity binding of RXR to *CLN1*, *CLN3*, *CLN6*, *CLN7*, *CLN8*, *CLN10*, and *CLN13*. Since PPARα and RXRα have been demonstrated to control the transcription of *CLN2* gene, our current promoter analysis findings highlight a possible treatment strategy for neuronal ceroid lipofuscinoses using agonists of PPARα and RXRα.

## Introduction

Neuronal ceroid lipofuscinosis (NCL), also known as Batten disease, comprise a category of inherited neurodegenerative disorders that primarily affect children and are characterized by the progressive accumulation of neuronal and extraneuronal autofluorescent storage material. Together, they represent the most predominant neurodegenerative disorders in children with age of onset varying from birth through young adulthood [1–3]. These fatal autosomal recessive disorders share clinical features characterized by progressive cognitive decline, deterioration of motor capabilities, retinopathy leading to blindness and epileptic seizures. Different forms of NCLs have a diverse range of biochemical etiology, disease symptoms, rate of disease progression and severity which makes the development of effective therapy for NCLs challenging. However, all NCLs exhibit common histopathological characteristics such as severe accumulation of autofluorescent ceroid lipopigments in various cells and neurodegeneration [2–5].

In 1826, Dr. Otto Christian Stengel, a physician in South-Eastern Norway published a report, ‘Account of a singular illness among four siblings in the vicinity of Røros’ narrating the first clinical description of NCL (Stangel, 1826) [6]. He had witnessed a local family with four children who showed unremarkable initial development and deterioration of sight. Eventually the disease resulted in blindness, speech impairment, progressive mental decline and epileptic seizures ultimately leading to premature death and was later identified to be similar to classic juvenile CLN3 disease [6, 7]. Since its discovery, various forms of the NCL disease have been identified and are also known as their eponyms. NCLs are collectively known as Batten disease after the British neurologist Frederick Batten who discovered two forms of the disease [7]. The term NCL was coined by Zeman and Dyken in 1969 [8]. The term ‘lipofuscin’ is derived from Greek ‘lipo’ meaning fat and latin ‘fucus’ meaning dark. Lipofuscins are yellowish brown, autofluorescent, electron dense materials that accumulate in post mitotic cells such as neurons, cells of retinal pigment epithelium, heart and skeletal muscle cells, and are considered most consistent morphological marker of aging [9].

## NCLs

### Pathophysiology of NCLs

In spite of their genetic heterogeneity, varied age of onset and distinct clinical outcomes, all forms of NCL share common pathological features i.e. accumulation of autofluorescent, materials in the cytoplasm of nerve cells as well as other cell types resulting in neuronal loss and gliosis. These storage materials were detected as be periodic acid-Schiff (PAS)- and Sudan black B-positive and resistant to lipid solvents [8]. The major components of the storage material were identified to be subunit c of the mitochondrial ATP synthase (SCMAS) or sphingolipid activator proteins (SAP) A and D [6]. The chemical nature of the predominant storage compound determines the ultrastructure of the tissue which can be visualized and confirmed by electron microscopy. While SAP accumulation has been observed to have an appearance known as Granular Osmiophilic Deposits, the storage of SCMAS has been found to form more variable patterns including curvilinear profiles, rectilinear complex and fingerprint profiles [6, 9].

### Classification of NCLs

Classically, NCLs were categorized according to the age of onset and grouped into infantile, late-infantile, juvenile, adult and were identified with their eponyms. At present, 14 genes have been implicated in NCL off which 13 have been identified, except for CLN9, which refers to a predicted locus without any identified mutations [2, 10]. A new nomenclature based on genetic origin and specific to phenotypic variances from different mutations has been recently developed. This classification system includes seven diagnostic axes i.e. affected *CLN* gene, precise genetic defect involving mutation, clinical phenotype (age at onset, presenting symptoms, and disease progression), molecular and cellular pathology involved, biomarkers, stage of functiona l impairment and any additional disabilities (genetic or environmental factors) [2, 11].

### Genes and proteins affected in NCLs

The proteins involved in different forms of NCLs have different intracellular localization and functions (for which it is known). Different forms of the disease originate from defects in lysosomal enzymes (CLN1, CLN2, CLN10, CLN13), transmembrane proteins (CLN3, CLN6, CLN7, CLN8), mutations in ATPase gene (CLN12) or potassium channels (CLN14) [1] and are briefly summarized in Table 1. The cellular functions of the NCL related proteins have been thoroughly discussed in the review by Kollmann et al. [12].

### Therapeutic approaches for NCLs

Currently, there is no curative therapy for most NCLs and treatments are targeted at managing the symptoms such as seizures, depression and behavioral abnormalities [13, 14]; however, several therapeutic approaches have shown promising results and are at different stages of clinical trial. Early diagnosis plays a vital role in managing NCL symptoms and could improve the quality of life of the patients. Initial laboratory studies, comprised of enzymatic tests, light microscopy and electron microscopic studies of the intracellular storage, are followed by appropriate molecular genetic confirmation of the NCL disease form [1, 9]. Some of therapeutic strategies for NCLs are targeted at replacing or correcting the defective protein by delivering the native form of the protein. The delivered corrected form of the protein needs to be sustainably expressed in adequate amount throughout the brain without any adverse toxicity. Although these pose significant challenge for designing novel therapy, restoration of 5–15 % of the regular enzyme function is sufficient to reestablish normal function of the protein in lysosomal storage disorders [14–16].

Enzyme replacement therapy (ERT), virus-mediated gene therapy, stem cell transplantation are being widely tested in animal models and human patients of NCL [17–25]. In ERT, a cross-correction strategy is used by administering recombinant form of the required protein into the brain of patients. Recently, ERT by intraventricular infusion of cerliponase alfa (recombinant human pro-TPP1) has been shown to reduce the impairment of locomotor functions and language impairment in children affected with CLN2 disease [26]. This is a huge leap towards the discovery of treatment for NCLs as cerliponase alfa is the first FDA-approved therapy for late infantile NCL or CLN2 disease; however, the treatment also presented adverse side effects including infection from the intraventricular device [26]. Moreover, the study involved repeated administration of intraventricular device which could be painful to the patients. Another concern with ERT is that since it requires receptor-mediated uptake of the normal enzyme inside the cell, it is potentially achievable only for NCLs that originate from a defective soluble lysosomal enzyme (i.e. PPT1, TPP1, Cathepsin D and Cathepsin F for CLN1, 2, 10 and 13 diseases respectively). Finally, purification of the recombinant protein and the repeated administration throughout the treatment duration makes ERT a very expensive mode of treatment.

In gene therapy, normal copy of the cDNA encoding the regular form of the protein is used for virus-mediated delivery into the CNS of the patients. Clinical trial using gene therapy for treatment of patients with CLN2 disease has demonstrated promising outcome [24]. CLN2 patients administered with an adeno-associated virus vector (AAV2) containing *CLN2* cDNA resulted in reduced rate of neurological decline compared to the untreated controls suggesting a slower rate of disease progression in the treated patients [24]. Although gene therapy constitutes a more sustained production of the required protein than other approaches, there are several challenges associated with it. For a successful gene therapy, sufficient amount of the required active protein has to be generated in order to reach the therapeutic levels for treatment. Another concern is that, the gene delivery needs to be able to target an extensive region of the brain for a long duration [20, 27]. Finally, like ERT, gene therapy could be invasive and painful for the children affected with NCL.

Small molecules including pharmacological chaperones, immune modulators are also being tested for the different forms of NCLs. For example, in case of CLN2 or late infantile NCL, several missense mutations are associated with abnormal folding of TPP1 protein which leads to an extended half-life of the pro form of the enzyme or aberrant trafficking to the lysosomes and ultimately, reduction in activity [28]. Nevertheless, treatments using chemical chaperones and permissive temperature have been demonstrated to be beneficial for some variants of TPP1 and thus could be further explored [28]. However, drugs modulating the different forms of NCLs are still understudied.

## Peroxisome proliferator-activated receptor (PPAR)

### PPARs and regulation of gene transcription by PPAR/RXR

PPAR is a subfamily of nuclear receptors and is comprised of ligand-inducible transcription factors that function as lipid sensors [29–32]. The human alpha (α), beta/delta (β/δ) and gamma (γ) isoforms of PPAR have distinct ligand specificity, tissue expression profile and physiological functions [33]. All PPARs control the expression of target genes via transcriptional regulation by forming heterodimers with transcriptional partner retinoid-X-receptors (RXRs). RXRs are nuclear receptors that are activated by retinoic acid and regulate several biological processes through transcriptional regulation. In the absence of stimulation by ligands, PPAR:RXR heterodimers are kept inactive via binding to corepressor molecules such as nuclear receptor co-repressor (NCoR), silencing mediator for retinoid and thyroid hormone receptor (SMRT) which, via direct interaction with Sin3 complex, recruits a multicomponent repressor complex [34–36]. Additionally, SMRT promotes the recruitment of histone deacetylases (HDACs) to the repressor complex. The corepressor complex containing HDACs suppresses the transcription of the target gene by causing histone deacetylation [37].

Upon stimulation and activation, in the nucleus, PPARs form heterodimers with RXRs and the PPAR:RXR heterodimer binds to the promoter of the target gene at cis-acting regulatory sequence known as peroxisome proliferator response element (PPRE). [30, 38]. Following ligand binding, a conformational change of the receptor leads to release of the co-repressors and facilitates recruitment of co-activators which enhance the transcription of target gene through histone acetylation/methylation and stabilization of basal transcription apparatus [30, 39]. Some of the coactivators associated with PPAR:RXR complex are: proteins exhibiting histone acetyl transferase (HAT) activity [CBP (cyclic-AMP responsive element binding protein (CREB)-binding protein)/P300, Steroid receptor coactivator (SRC) family of proteins] [40–45], proteins involved in recruitment of coactivators [PGC-1α, PGC-1β] [46, 47], chromatin remodeling complex that mobilizes nucleosomes in a ATP-dependent manner [SWI/SNF] [48, 49], helicases that facilitate histone displacement and sliding of nucleosomes [PRIC285, PRIC320] [50, 51].

The alpha isoform of PPAR (PPARα), plays a key role in regulation of energy homeostasis via induction of fatty acid and cholesterol metabolism and lowering of serum triglyceride content [29, 52]. Some of the prototype PPAR α agonists are lipid-lowering fibrate drugs (gemfibrozil, fenofibrate), WY14643 and GW7647 [53, 54]. The protein PPARα is composed of multiple functional domains: a zinc finger- containing highly conserved DNA binding domain (DBD) that is responsible for binding to the conserved PPRE site on the promoter of target gene, a hinge region, C-terminal ligand-binding domain (LBD) containing helices that form a large ligand binding pocket, E/F domain for dimerization with RXR and ligand-dependent transactivation of the receptor, N-terminal domain for ligand-independent receptor regulation [52, 55–57].

### Potential regulation of CLN genes by PPARα and RXRα

Previously, we have demonstrated that PPARα agonist gemfibrozil and fenofibrate enhanced the expression of the CLN2 gene via PPARα/RXRα transcriptional complex and enhanced TPP1 protein activity in mouse primary brain cells [58]. Hence, in this study, we investigated if other genes involved in NCLs could potentially be regulated by PPARα and/or RXRα. Using the MatInspector program in the Genomatix (Intrexon Bioinformatics Germany) software [59], we searched the promoter region of all 14 genes that are affected in NCLs for potential binding site of PPARα (PERO) and RXRα (RXRF). The human sequence of the corresponding gene (“Gene ID” in Tables 2 and 3) was obtained from Genomatix and the different transcripts were color labeled and grouped as specified by Genomatix: “Gold” corresponding to “experimentally verified 5′ complete transcript”, “Silver” corresponding to “transcript with 5′ end confirmed by PromoterInspector prediction” and “Bronze” corresponding to “annotated transcript, no confirmation for 5′ completeness”. The transcript selection conditions were: always select transcripts labeled as “Gold” whenever available, followed by selection of transcripts labeled as “Silver” (if “Gold” is not available) and lastly “Bronze” (if both “Gold” and “Silver” are not available). All of the transcripts that were selected for promoter search were fully or partially “gold”. Following selection of a particular transcript (“Sequence ID” in table), the promoter region of the gene was searched for PERO and RXRF and the total number of sites were documented. The matrix scores were used for determining the potential binding of the PPARs and RXRs to the promoter of the corresponding gene and described as matrix score (<0.75): “Negligible”, (0.75–0.80): “Low” affinity, (0.80–0.85): “Medium” affinity and (>0.85): “High” affinity binding.

Our analysis for the presence of matrix PERO showed that out of 14 NCL related genes, 9 have “High” affinity (CLN 2, CLN 3, CLN 4, CLN 5, CLN 7, CLN 10, CLN 11, CLN 12, CLN 14), 2 have “Medium” affinity (CLN 8, CLN 13), 1 has “Low” affinity (CLN 1) and 2 have “Negligible” affinity (CLN 6, CLN 9) for binding PPAR transcription factors. Similarly, our analysis for the matrix RXRF showed that 7 genes have “High” affinity (CLN 1, CLN 3, CLN 6, CLN 7, CLN 8, CLN 10, CLN 13), 3 genes have “Medium” affinity (CLN 2, CLN 5, CLN 11), 3 genes have “Low” affinity (CLN 4, CLN 12, CLN 14) and 1 gene has “Negligible” affinity for binding of RXR transcription factors (CLN 9).

### PPARα and RXR agonists as potential NCL therapeutic agents

**Figure 1:**
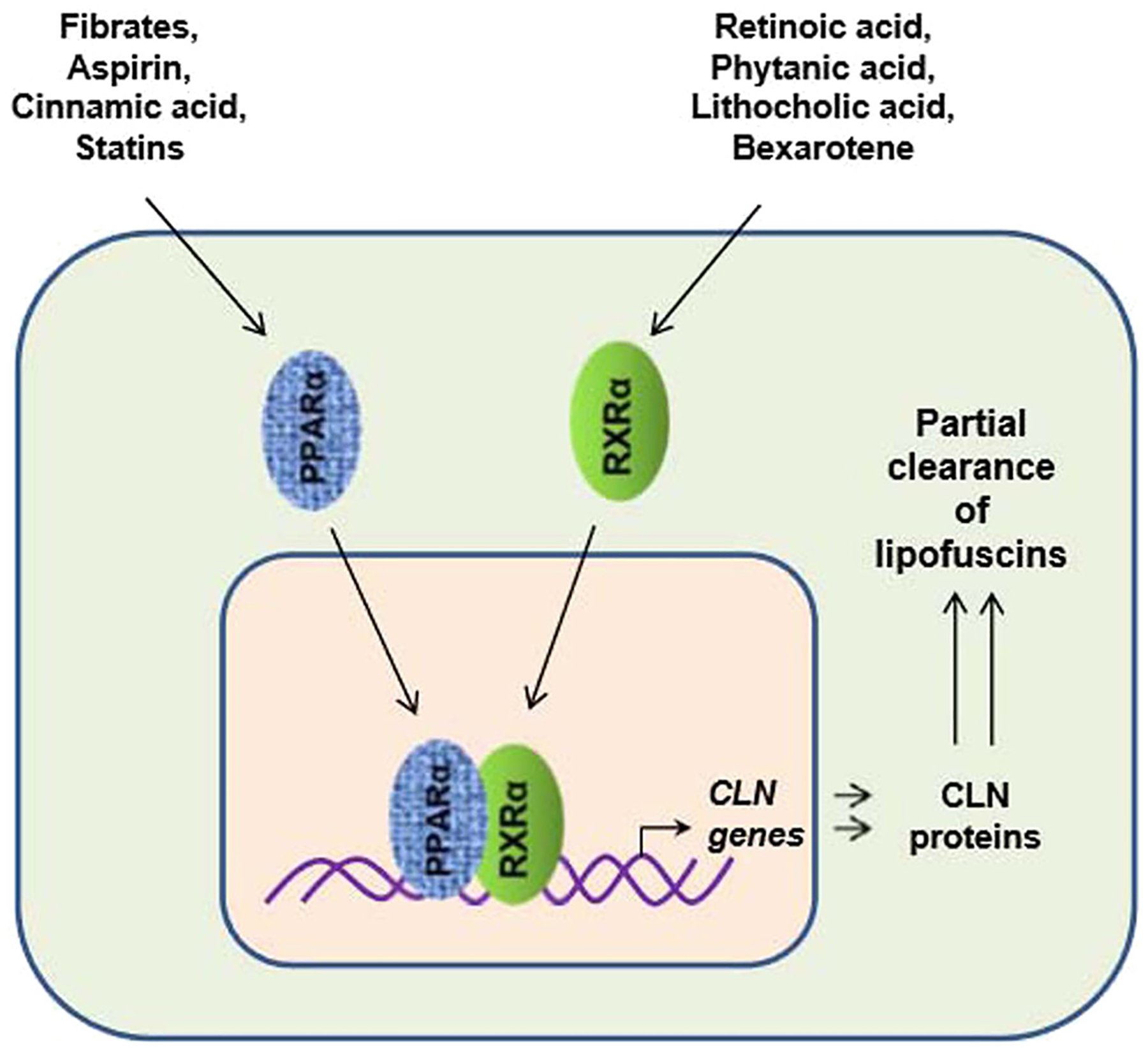
Proposed model for the stimulation of residual normal *CLN* genes and partial clearance of lipofuscins in NCLs via activation of the PPARα:RXRα pathway.

Growing evidence point towards a beneficial role of PPARα in lysosomal functions. Gemfibrozil, a prototype PPARα agonist and FDA-approved drug for hyperlipidemia, has been demonstrated to induce lysosomal biogenesis in mouse primary brain cells via upregulating the master regulator Transcription factor EB (TFEB) [60]. Importantly, a study reported that a combination of gemfibrozil and all transretinoic acid upregulate the TPP1 expression in mouse primary brain cells through transcriptional upregulation of *CLN2* gene and therefore has important implications for the treatment of LINCL or CLN2 disease [58]. Moreover, CLN1 and CLN3 mRNA expressions were increased in mouse primary astrocytes following gemfibrozil treatment [58]. Furthermore, *in vivo* gemfibrozil treatment also enhanced the TPP1 expression in the cortex and nigra of mouse in a PPARα-dependent manner [58]. The level of the enzyme TPP1 was also increased by gemfibrozil and all-trans retinoic acid (ATRA) in mouse primary brain cells [58]. Moreover, oral administration of gemfibrozil in a mouse model for LINCL (CLN2^−/−^) lowered the storage material (SCMAS), enhanced longevity and motor activity of the mice [61]. Therefore, gemfibrozil along with ATRA could be beneficial for treatment of NCLs. Moreover, we have demonstrated that widely-used drugs such as statins [62] and aspirin [63–65] and naturally occurring cinnamic acid [66] are also capable of binding and activating PPARα. In addition to retinoic acid, many different druggable compounds such as bexarotene, phytanic acid, lithocholic acid, etc. have also been found to activate RXRα [67]. Therefore, different combinations of PPARα and RXRα agonists (Figure 1) may be tried to reduce storage materials in NCLs.

The two mechanism of actions by which PPARα and RXRα could regulate the expression of these NCL genes are: (1) via transcriptionally upregulating the expression of the NCL genes; (2) via upregulating TFEB which can further bind to the CLEAR (coordinated lysosomal expression and regulation) element on lysosomal genes and induce lysosomal biogenesis. Interestingly, a study in a murine model of LINCL suggests that achievement of as low as 6 % TPP1 functionality in the CNS could have beneficial effects [68]. Although NCLs arise from mutations in the affected gene, many evidences indicate that residual function of the normal protein is found in disease model or patients. For example, in case of LINCL, residual activity of the TPP1 enzyme has been found to be retained in patients [4, 28, 69]. Therefore, if a few copies of the normal gene are retained in the patients, PPARα and RXRα agonists could potentially enhance the transcription and production of the normal protein. Alternatively, this strategy could be used as an adjunct therapy along with other techniques like gene therapy to enhance the production and efficacy of the required protein. The normal cDNA delivered via gene therapy could further be transcriptionally regulated to produce more amount of the therapeutic protein and a stronger effect.

Our results show that many of the NCL-affected genes harbor one or more PERO and/or RXRF in their promoter region and could potentially be regulated by agonists of the transcription factors PPARα and RXRα. There are several advantages of this drug-based treatment strategy. Firstly, many FDA-approved agonists of PPARα (fibrate drugs, gemfibrozil and fenofibrate) are already available and could be repurposed for NCL. Another advantage would be that these drugs could be taken orally which is the least painful mode of treatment. Gemfibrozil has been tested in children before in several studies and was concluded to be safe for use in children [70–72]. Finally, these drugs are much more economical than some of the other available treatment options like ERT.

## Conclusions

At present, there is no cure for NCLs and treatments are directed towards symptomatic relief only. Therefore, there is a necessity for innovative therapies that halt or slower the disease progression and improve the life of the children affected by this fatal disorder. Our preliminary analysis suggests that PPARα and RXRα agonists could potentially regulate several NCL related genes (Figure 1). While *CLN2*, *CLN3*, *CLN4*, *CLN5, CLN7*, *CLN10*, *CLN11*, *CLN12*, and *CLN14* exhibit high affinity binding of PPAR, *CLN1*, *CLN3*, *CLN6*, *CLN7*, *CLN8*, *CLN10*, and *CLN13* display high affinity binding for RXR. Therefore, agonists of PPARα and RXRα, alone and together, could be evaluated further through *in vitro* and *in vivo* studies. For example, recently we have seen that activation of PPARα by gemfibrozil leads to neuroprotection in a mouse model of Juvenile Neuronal Ceroid Lipofuscinosis [73]. In summary, this review highlights a potential role for PPARα and RXRα in the transcriptional regulation of different NCL associated genes.

## Figures and Tables

**Table 1:** Genes and proteins affected in NCLs.

Disease	Phenotype MIM number	Gene symbol	Locus	Gene/locus MIM number	Protein name
CLN1	#256730	PPT1	1p34.2	*600722	Palmitoyl-protein thioesterase 1
CLN2	#204500	TPP1	11p15.4	*607998	Tripeptidyl peptidase 1
CLN3	#204200	CLN3	16p12.1	*607042	CLN3
CLN4	#162350	DNAJC5	20q13.33	*611203	Cysteine string protein alpha
CLN5	#256731	CLN5	13q22.3	*608102	CLN5
CLN6	#601780	CLN6	15q23	*606725	CLN6
CLN7	#610951	MFSD8	4q28.2	*611124	Major facilitator superfamily domain-containing protein 8
CLN8	#600143	CLN8	8p23.3	*607837	CLN8
CLN9	%609055	n/a	n/a	n/a	Unknown
CLN10	#610127	CTSD	11p15.5	*116840	Cathepsin D
CLN11	#614706	GRN	17q21.31	*138945	Progranulin
CLN12	#606693	ATP13A2	1p36.13	*610513	ATPase type13A2
CLN13	#615362	CTSF	11q13.2	*603539	Cathepsin F
CLN14	#611726	KCTD7	7q11.21	*611725	Potassium channel tetramerization domain containing protein 7

The different genes involved in NCLs are listed along with their MIM numbers and encoded proteins.

**Table 2:** PERO on human *CLN* gene promoters.

Disease	Gene symbol	Gene ID	Sequence ID	Total no. of PERO	Region	Matrix	Matrix score	Predicted recruitment
CLN1	*PPT1*	5538	GXP_260673	1	^1016^ gcagtacagggcagTGGTgaaga ^1038^	PPAR/RXR heterodimers, DR1 sites	0.792	Medium
CLN2	*TPP1*	1200	GXP_6730504	4	^885^ gcttgactggccAGAGgggagaa ^907^	PPARγ	0.888	Medium
CLN3	*CLN3*	1201	GXP_7532773	3	^1741^ aaggaggaggggAAAGgtgaggc ^1763^	PPARγ	0.952	High
CLN4	*DNAJC5*	80331	GXP_98072	2	^579^ ccagggttgagcAAATgtcagtc ^601^	PPARγ, DR1 sites	0.856	Medium
CLN5	*CLN5*	1203	GXP_7529957	1	^828^ tgggatggggtaAAAGttcggtg ^850^	PPARγ	0.890	Medium
CLN6	*CLN6*	54982	GXP_227470	n/a	n/a	n/a	n/a	n/a
CLN7	*MFSD8*	256471	GXP_263769	2	^479^ cacaaatgacacAAAGatcagaa ^501^	PPARγ, DR1 sites	0.855	Medium
CLN8	*CLN8*	2055	GXP_1504822	1	^147^ tttgccccttaaAAAGgttaact ^169^	PPARγ, DR1 sites	0.839	Medium
CLN9	*n/a*	*n/a*	*n/a*	*n/a*	*n/a*	*n/a*	*n/a*	*n/a*
CLN10	*CTSD*	1509	GXP_7526123	1	^1022^ aagctgggaggcAAAGgctacaa ^1044^	PPARγ, DR1 sites	0.863	Medium
CLN11	*GRN*	2896	GXP_6035231	4	^386^ agggtgcgggagAAAGtgcaaga ^408^	PPARγ	0.896	Medium
CLN12	*ATP13A2*	23400	GXP_42195	2	^394^ attgaccaggagAAAGgcctggc ^416^	PPARγ	0.905	High
CLN13	*CTSF*	8722	GXP_50540	1	^864^ caagcacgtgatAGAGgtcagtg ^886^	PPARγ, DR1 sites	0.851	Medium
CLN14	*KCTD7*	27342	GXP_40481	2	^231^ tagaatgtggtcAAAGaccaagt ^253^	PPARγ	0.894	Medium

Promoter regions of all 14 NCL-related genes were searched using the MatInspector program in the Genomatix software. The number of total PERO sites in the promoter region is listed along with a prediction for binding affinity of PPARs.

**Table 3:** RXRF on human *CLN* gene promoters.

Disease	Gene symbol	Gene ID	Sequence ID	Total no. of RXRF	Region	Matrix	Matrix score	Predicted recruitment
CLN1	*PPT1*	5538	GXP_260673	5	^585^ tttttgatgtTTTGttgaaaaaaaa ^609^	RXR heterodimer binding sites	0.882	Medium
CLN2	*TPP1*	1200	GXP_6730504	5	^888^ tgactggccagAGGGgagaatccgg ^912^	RAR/ RXR heterodimer, DR1 sites	0.812	Medium
CLN3	*CLN3*	1201	GXP_7532773	6	^1631^ gattaggggtacGAGGacgcatggt ^1655^	VDR/RXR heterodimer, DR3 sites	0.895	Medium
CLN4	*DNAJC5*	80331	GXP_98072	1	^250^ tgaccgcactgaactcAGGTaaacg ^274^	THRβ (ER6 - everted repeat, spacer 6)	0.752	Medium
CLN5	*CLN5*	1203	GXP_7529957	5	^899^ ggcaaGGACaggtcaattcataaaa^923^	Constitutive AR/ RXR heterodimer, DR4 sites	0.809	Medium
CLN6	*CLN6*	54982	GXP_227470	5	^761^ ctccgggGTTCataggggtcacggg ^785^	Bipartite binding site of VDR/RXR heterodimers, DR3 sites	0.885	Medium
CLN7	*MFSD8*	256471	GXP_263769	6	^976^ tgagtgacatTTTGcaaggctgagg ^1000^	RARγ, homodimer DR2 binding site	0.947	High
CLN8	*CLN8*	2055	GXP_1504822	5	^643^ atcagGGTCacctgtgctcagccat ^667^	Nuclear receptor involved in the regulation of lipid homeostasis, DR4 element	0.911	High
CLN9	*n/a*	*n/a*	*n/a*	*n/a*	*n/a*	*n/a*	*n/a*	*n/a*
CLN10	*CTSD*	1509	GXP_7526123	5	^768^ aaggagggctgtGAGGccattgtgg ^792^	VDR/RXR heterodimer, DR3 sites	0.902	High
CLN11	*GRN*	2896	GXP_6035231	5	^830^ gtggagggaagtGGGGgcagagttg ^854^	VDR/RXR heterodimer, DR3 sites	0.854	Medium
CLN12	*ATP13A2*	23400	GXP_42195	2	^165^ ggcgcgatctcaGCTCactgcaccc^189^	RXR homodimer, DR1 sites	0.780	Medium
CLN13	*CTSF*	8722	GXP_50540	3	^867^ gcacgtgatagaGGTCagtgactag ^891^	RXRα homodimer, DR1 sites	0.876	Medium
CLN14	*KCTD7*	27342	GXP_40481	5	^201^ ttagagagcataGTTCaaaatcagg ^225^	RXR homodimer, DR1 sites	0.795	Medium

The promoter regions of all 14 *CLN* genes were searched using the MatInspector program in the Genomatix software. The number of total RXRF sites in the promoter region is listed along with a prediction for binding affinity of RXRs. AR, androgen receptor; RAR, retinoic acid receptor; RXR, reninoid X receptor; VDR, vitamin D receptor; THR, thyroid hormone receptor.

## Data Availability

All data are presented here.
